# Antioxidant Intake and Ovarian Reserve in Women Attending a Fertility Center

**DOI:** 10.3390/nu17030554

**Published:** 2025-01-31

**Authors:** Ana B. Maldonado-Cárceles, Irene Souter, Ming-Chieh Li, Makiko Mitsunami, Irene Dimitriadis, Jennifer B. Ford, Lidia Mínguez-Alarcón, Jorge E. Chavarro

**Affiliations:** 1 Department of Nutrition, Harvard T.H. Chan School of Public Health, 655 Huntington Avenue, Boston, MA 02115, USA; anab.maldonadocarceles@gmail.com (A.B.M.-C.); mmitsunami@hsph.harvard.edu (M.M.); 2 Department of Health and Social Sciences, Division of Preventive Medicine and Public Health, University of Murcia School of Medicine, 30100 Espinardo, Murcia, Spain; 3 Fertility Center, Vincent Department of Obstetrics and Gynecology, Massachusetts General Hospital and Harvard Medical School, 32 Fruit Street, Suite 10A, Boston, MA 02114, USA; isouter@mgh.harvard.edu (I.S.); idimitriadis@partners.org (I.D.); 4 Department of Health Promotion and Health Education, National Taiwan Normal University College of Education, Taipei 106, Taiwan; mingchiehli@ntnu.edu.tw; 5 Department of Environmental Health, Harvard T.H. Chan School of Public Health, 655 Huntington Avenue, Boston, MA 02115, USA; jford@hsph.harvard.edu (J.B.F.); lminguez@hsph.harvard.edu (L.M.-A.); 6 Channing Division of Network Medicine, Department of Medicine, Brigham and Women’s Hospital and Harvard Medical School, 181 Longwood Avenue, Boston, MA 02115, USA; 7 Department of Epidemiology, Harvard T.H. Chan School of Public Health, 655 Huntington Avenue, Boston, MA 02115, USA

**Keywords:** antioxidants, antral follicle count, female fertility, lycopene, retinol, ovarian reserve

## Abstract

Background/Objectives: The aim of this study was to investigate the association between antioxidant intake and antral follicle count (AFC), a marker of ovarian reserve, in women attending a fertility clinic. Methods: We conducted an observational study with 567 women undergoing infertility evaluation at the Massachusetts General Hospital Fertility Center, who were enrolled in the Environment and Reproductive Health (EARTH) study. Participants filled out the lifestyle and health questionnaires and a validated food frequency questionnaire (FFQ) for assessing habitual dietary intake and underwent a transvaginal ultrasound to measure AFC. Intake of nutrients with direct antioxidant capacity (vitamin A, C, and E and carotenoids) and intake of antioxidant food sources were estimated from the FFQ. Adjusted Poisson regression models were fitted to assess the relationships between antioxidants and AFC while adjusting for potential confounders. Non-linearity was assessed with restricted cubic splines. Results: The median (interquartile range) age and AFC of participants were 35.0 (32.0–38.0) years and 13 (9–18), respectively. Our findings revealed a non-linear association between lycopene intake and AFC. There was a positive linear association with the highest AFC among women consuming approximately 6000 mcg/day of lycopene (*p* for non-linearity = 0.003). An inverse association was observed between retinol intake, predominantly from dairy foods, and AFC among women aged under 35 years (*p*-trend < 0.001 and 0.01, respectively). Conclusions: Our findings suggest that lycopene intake might influence the ovarian reserve in fertility patients. The observed inverse association with retinol, if confirmed, may reflect biological mechanisms different from oxidative stress. The underlying mechanisms of these associations remain to be elucidated and warrant further investigation.

## 1. Introduction

Oxidative stress, the imbalance between reactive oxygen species (ROS) an antioxidant activity, is associated with several human health conditions, including female reproductive disorders [1,2]. In the ovary, higher levels of ROS and reduced antioxidant content are associated with advanced female age, indicating a decreased capacity to scavenge ROS as age progresses [3,4]. Oxidative stress directly damages the intraovarian environment, which seriously impacts ovulation, meiosis, luteolysis, and folliculogenesis, including granulosa cell apoptosis and follicular atresia, culminating in the ovarian aging process [3,4,5,6,7]. Ovarian aging is characterized by a diminished ovarian reserve with the decline in both the quality and quantity of oocytes, which is an important cause of infertility and poorer success in assisted reproductive technology outcomes [4,8]. Therefore, alleviating oxidative stress in the ovaries is an important entry point for delaying ovarian aging [4].

Antioxidant intake has been proposed to improve the oxidative stress state and enhance ovarian function as potential therapeutic options to delay aging and improve reproductive outcomes, alleviating the apoptotic loss of ovarian follicles due to oxidative stress [7,9,10]. Vitamins A, C, and E and carotenoids possess antioxidant properties that include scavenging free radicals to diminish DNA damage, protecting against lipid peroxidation, and defending cell membranes from injury [11,12]. Various randomized controlled trials (RCTs) have evaluated the effects of antioxidants, including vitamins A, C, and E, on female reproductive outcomes [13,14]. For example, two RCTs demonstrated that vitamins C and E combined improved outcomes in endometriosis compared to placebo. Vitamin C alone also showed beneficial effects in women with endometriosis-associated infertility undergoing assisted reproductive treatment (ART) [12]. In patients with polycystic ovary syndrome (PCOS), supplementation with a mixture of antioxidants, including vitamins A, C, and E, significantly improved pregnancy rates, live births, and oxidative stress markers, as well as serum levels of these vitamins and minerals, compared to controls [15]. However, in women with unexplained infertility, supplementation with multivitamins and minerals containing vitamins A, C, and E did not yield beneficial effects on ART outcomes compared to placebo [16]. Interpreting the existing RCTs is challenging because most studies evaluate combinations of micronutrients with varying treatment regimens and administration methods (oral supplementation or direct addition during oocyte collection in vitro). Additionally, these combinations often include substances without direct antioxidant activity, making it difficult to isolate the effects of specific nutrients and standardize clinical applications [10,14].

Despite the wealth of research on antioxidants and female fertility [13,14], as well as their widespread use in gynecological clinical practice [12], their role in improving female fertility, and particularly in relation to the ovarian reserve, remains poorly understood [13], partly due to the paucity of epidemiological studies in women examining the consumption of these antioxidants and their relation to biomarkers of ovarian reserve. An RCT involving 70 women with premature ovarian insufficiency (POI) assessed the effect of supplementation with vitamin E (400 IU for 90 days) and selenium on antral follicle count (AFC) and anti-Mullerian hormone (AMH) levels, reporting increases in both markers in the intervention group. The authors suggested that this supplementation could reduce oxidative stress-induced damage in POI [17]. Studies in animal models showed that micronutrients with direct antioxidant capacity, such as vitamins A [6,18], C [19], and E [6,19,20], as well as carotenoids [3,21], have been linked to more favorable markers of pro-oxidants and antioxidants [3,6,21] and improvements in the quantity of oocytes [3,18,19,20,21]. Identifying the role of specific antioxidants and their effective doses is important for designing future interventional studies [14]. Therefore, we investigated the relationships between the intake of micronutrients with direct antioxidant capacity, specifically vitamins A, E, and C and carotenoids, and AFC, a marker of ovarian reserve.

## 2. Materials and Methods

### 2.1. Study Population

Participants were invited to participate in the Environment and Reproductive Health (EARTH) Study, a prospective cohort established in 2004 that recruited subfertile couples presenting to the Massachusetts General Hospital (MGH) Fertility Center and aimed to investigate environmental and dietary factors in relation to human fertility [22]. At enrollment, all participants completed a general health questionnaire with demographics, lifestyle, and medical and reproductive history and underwent an ultrasound exam. Starting in 2007, a semiquantitative food frequency questionnaire (FFQ) was introduced as part of the baseline data collection to assess usual dietary habits. Of the 781 scans from women who completed the FFQ, we excluded scans of women using leuprolide (*n* = 25), women with a history of oophorectomy (*n* = 5), women with a diagnosis of polycystic ovary syndrome (*n* = 49), scans where it was difficult to visualize the ovary (*n* = 7), scans with incomplete AFC data (*n* = 8), repeated scans from the same women (*n* = 85), women who completed the FFQ more than a year after their transvaginal ultrasound (*n* = 22), as well as women who reported implausible total energy intake (<500 and >3500 kcal/day) (*n* = 13), leaving a final sample size of 567 women. Ethical approval was obtained from the Human Studies Institutional Review Boards of the MGH and the Harvard T. H. Chan School of Public Health, and all participants provided written informed consent.

### 2.2. Antral Follicle Count Measurement

The women included in this study underwent a standard infertility work-up, which included the ultrasonographic determination of the AFC for ovarian reserve evaluation. AFC was ascertained by one of the MGH Reproductive Endocrinology and Infertility physicians using transvaginal ultrasound. AFC measurements were performed after ensuring that women were in the early follicular phase, following the standard protocol from the MGH Center and in accordance with the recommendations of the American Society for Reproductive Medicine Practice Committee [23]. When performed in experienced centers, AFC has low intercycle variability and high interobserver reliability. No fertility medications were used in the cycle prior to the ultrasonographic assessment.

### 2.3. Antioxidant Intake Assessment

Diet was assessed using an extensively validated [24,25] semi-quantitative FFQ. In brief, this questionnaire requested information on the participants’ usual intake of foods and beverages during the previous year in 9 response categories per food item, ranging from never or less than once a month to ≥6 times per day. Multivitamin and supplement users were also asked to specify the brand of the multivitamin or supplement, the dose, and frequency of use. Nutrient values for each food item were compiled from the US Department of Agriculture National Nutrient Database [26] and food manufacturers. Estimates of intake for nutrients with a direct antioxidant capacity (vitamin A, C, and E, retinol, and carotenoids (α-carotene, β-carotene, β-cryptoxanthin, lycopene, lutein, and zeaxanthin)) were obtained by summing the nutrient content of each of these nutrients across all items in the FFQ, weighing the intake of each food item with a weight proportional to the frequency of its use. Vitamin A intake is presented as retinol activity equivalents (RAE; micrograms per day) and includes the intake of retinol (preformed vitamin A) and of provitamin A carotenoids weighed by their retinol-forming capacity [27,28]. Nutrient intakes were adjusted for total energy intake using the nutrient residual method [29].

### 2.4. Statistical Analysis

Pair-wise Spearman correlation coefficients between the intake of all antioxidants were calculated. Antioxidants were divided into four groups according to quartiles of intake. Kruskal–Wallis and χ2 tests for continuous and categorical variables were performed to detect differences in participant characteristics across quartiles of antioxidants intake. A total of 22 (22; 4%) of the 567 women included in the analysis had an AFC > 30. To reduce the influence of these high values, we truncated AFC at 30. We estimated predicted AFC marginal means and 95% confidence intervals of each quartile of antioxidants intake using multivariate generalized linear models with Poisson distribution and log-link function using the overall sample means of continuous covariates for all quartiles and the reference categories according to their frequencies for categorical variables. These estimates were back-exponentiated so that we could show them in the original count scale. To test for linear trends across quartiles of antioxidants intake, we assigned the median value to each category and modeled this variable as a continuous variable. We examined the possibly non-linear relation between antioxidant intake and AFC non-parametrically with restricted cubic splines [30,31] which used the likelihood ratio test, comparing the model with only the linear term to the model with the linear and the cubic spline terms. Specifically, the procedure to test the non-linear relationship compares the log-likelihood of the model containing the new variable (spline term) with the log-likelihood of a model containing the predictor as a linear variable. This procedure involves several steps. Each step starts with a “base” model. For the first step, the “base” model includes the linear term and all the adjusters. For subsequent steps, the “base” model includes the above plus whatever spline variables are in the model by the end of the step. Likelihood ratio tests are used at each step to determine whether adding or removing a spline term significantly improves the model fit [31].

Potential confounders were selected based on prior knowledge and descriptive statistics from our study population. Using these criteria, the final model included age (continuous), smoking status (never smoked, ever smoked), race (White/Caucasian, other), body mass index (BMI) (continuous), total energy intake (continuous), physical activity (continuous), vitamin B12 (continuous), folate (continuous), caffeine (continuous), and intakes of the remaining antioxidants (continuous). Interactions by demographic characteristics (age, BMI, and smoking status) were tested by including cross-product terms in the final multivariate model. Analyses were performed using the statistical software SAS v. 9.4 (SAS Institute Inc., Cary, NC, USA). All statistical tests of the data were two-tailed at a significance level of <0.05.

## 3. Results

The 567 women in our study were predominantly Caucasian (83%), had never smoked (75%), had a college degree or higher (93%), and had a median (IQR) age at study entry of 35.0 (32.0–38.0) years, an AFC of 13 (9–18), and a BMI of 23.2 (21.3–26.1) kg/m^2^ (Table 1). Associations between antioxidant intakes and baseline patient characteristics are presented in Supplementary Tables S1a,b. A lower intake of vitamin A was related to higher calorie intake, and participants with higher carotenoid intake were less likely to have had a prior infertility treatment. Women who were more physically active reported higher intakes of vitamin A, β-carotene, lutein, and zeaxanthin. Women with a high intake of retinol were more likely to have a male infertility diagnosis, and Caucasian women reported a lower intake of α-carotene and β-cryptoxanthin. Higher intakes of total vitamins A, C, and E, retinol, total carotenoids, β-carotene, and β-cryptoxanthin were related to higher intakes of folate and vitamin B12, and higher intakes of vitamin A and α-carotene were related to a lower intake of caffeine. Women with higher consumption of lycopene were younger and reported higher folate intakes. Subjects with a higher consumption of lutein and zeaxanthin were more likely to have had a prior fertility exam, a prior infertility treatment, and a higher intake of vitamin B12. In general, antioxidants were weakly correlated to each other (Supplementary Table S2). Lycopene was the antioxidant with the lowest correlations with all other antioxidants, ranging between r = 0.02 with vitamin E and r = 0.10 with α-carotene.

**Figure 1:**
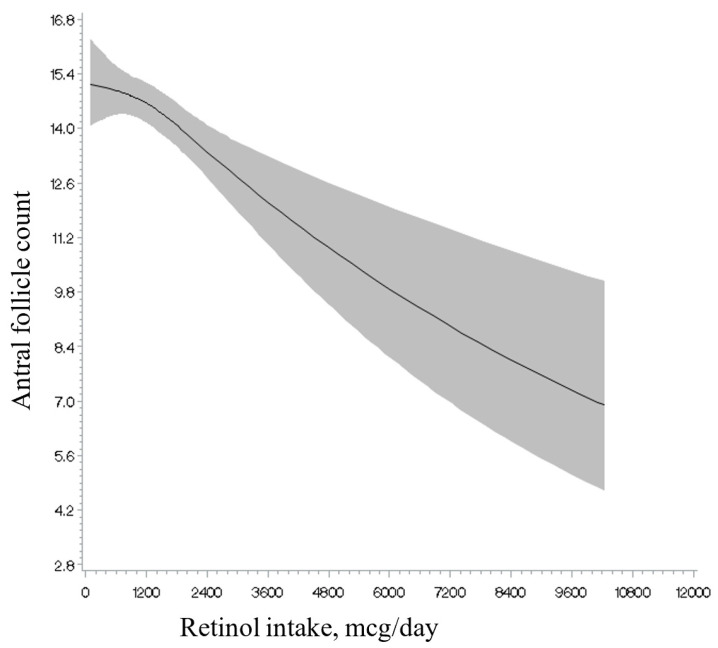
Restricted cubic spline plot of the association between retinol intake and antral follicle count among 567 women in the EARTH study after multivariable adjustment. *p*-value for non-linearity = 0.23.

**Figure 2:**
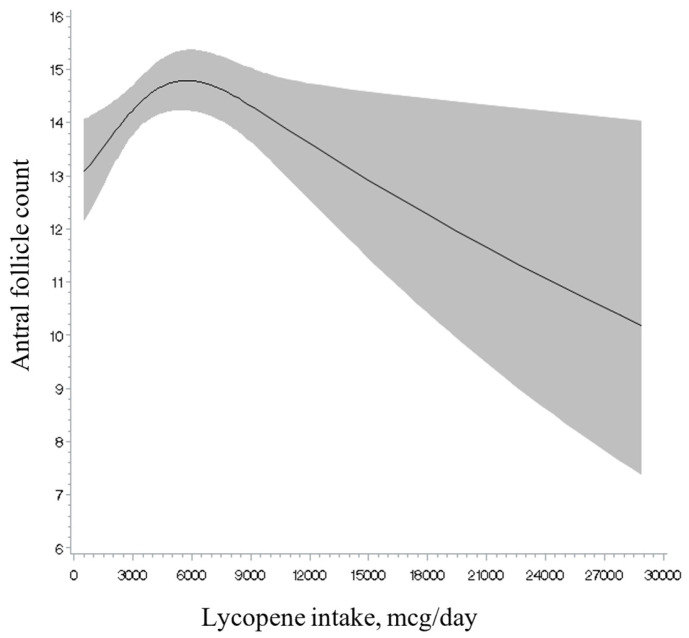
Restricted cubic spline plot of the association between lycopene intake and antral follicle count among 567 women in the EARTH study after multivariable adjustment. *p*-value for non-linearity = 0.003. After restriction at the 95th percentile of lycopene intake from food distribution, the *p*-value for non-linearity was 0.003.

**Figure 3:**
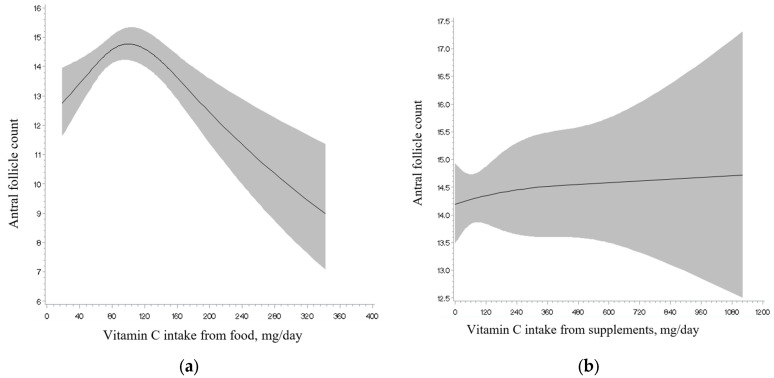
Restricted cubic spline plot of the associations between vitamin C intake from food source (**a**), supplement source (**b**) and antral follicle count among 567 women in the EARTH study after multivariable adjustment. *p*-value for non-linearity was <0.001 for food source, and 0.82 for supplement source. After restriction at the 95th percentile of vitamin C intake from food distribution, the *p*-value for non-linearity was 0.15 for food source.

Total intakes of vitamins A, C, and E and retinol, as well as of total and individual carotenoids, were unrelated to AFC (Table 2). Analyses separating intake from foods and intake from supplements showed a similar result (Supplementary Table S3). Non-linearity tests (Figure 1, Figure 2 and Figure 3; Supplementary Figures S1–S14) were not significant for the majority of the antioxidants examined (e.g., retinol intake; see Figure 1), with the exception of lycopene intake (Figure 2), and vitamin C (Figure 3) and E intake from food in relation to AFC (*p* for non-linearity = 0.003, <0.001 and 0.04, respectively). Specifically, we observed an inverse U-shaped association with the highest AFC among women with a consumption at approximately 6000 mcg/day of lycopene intake and 100 mg/day of vitamin C from food and a U-shaped relationship with the lowest AFC at approximately 10 mg/day of vitamin E from food. However, only lycopene intake remained statistically significant after excluding outliers above the 95th percentile of the intake distribution (*p* for non-linearity = 0.003). The main food sources for lycopene intake in our population (>1%) were tomato-containing products. Specifically, these included tomato sauce (54.5%), tomato juice (16.1%), salsa picante or taco sauce (13.0%), ketchup or red chili sauce (3%), pizza (2%), and tomatoes (2%) (data not shown).

Last, we evaluated whether the association observed between antioxidant intake and AFC was modified by age, BMI, or smoking. We found that the intake of retinol was related to lower AFC among women under 35 years of age (*p*-interaction < 0.001) (Supplemental Table S4). Although other interactions were identified, those findings were no longer significant after minor changes or relevant cutoff values (Data not shown). As we observed a high variability in the top quartile of retinol intake range (1554.1–10,228.5 mcg/day), we additionally assessed the association with AFC restricting total distribution below the threshold of two (3021.6 mcg/day) and three (3902.1 mcg/day) standard deviations (SD). The relationships after restriction to two and three SDs were nearly similar to those observed with total distribution (*p*-interactions and *p*-trends < 0.001) (data not shown). We also examined the associations between the intake of the main food sources of retinol (dairy products, organ meat, seafood, and cereals) [27,28] and AFC among women under 35 years old (251 women). In this food-based analysis, only the intake of dairy products was inversely related to AFC (*p*-trend = 0.01) (data not shown). We then examined whether other nutrient components in dairy, such as saturated and trans fatty acids, might be responsible of the observed associations with retinol (interaction effect of age on retinol and AFC, as well as dairy product intake and AFC, in the food analysis among women under 35 years). Both models remained similar after adding saturated fatty acids (*p*-interaction < 0.001 and *p*-trend = 0.008, respectively) and trans fatty acids (*p*-interaction < 0.001 and *p*-trend = 0.01, respectively), one at a time, to the set of covariates.

## 4. Discussion

In this cohort of 567 women from a fertility clinic, we found a positive association between lycopene intake and AFC, which seemed to reach a maximum at intake levels of 6000 mcg/day (~1.6 times the median levels of consumption). The main food sources of lycopene among our participants were tomato-containing products, accounting for more than 95% of the total intake. Additionally, we found the intake of retinol to be inversely associated with this marker among women aged under 35 years, which was solely driven by the intake of dairy foods. The association patterns observed for retinol and dairy food sources suggest that this relationship may not necessarily reflect biologically relevant retinol activity in the ovarian reserve. These results, if confirmed among other women, may be used to improve women’s fertility potential by consuming certain types of nutrients and foods.

Previous studies examining the association between lycopene, including its main source (tomato and tomato-based products), and ovarian reserve or ovarian senescence are sparse. Moslehi and colleagues [9], who followed a cohort of healthy women aged 20–50, found that the annual rate of decline was not related to tomato consumption. However, tomato-containing products such as tomato sauce were not reported, even though they are ideal sources of lycopene due to their high concentration of this carotenoid, which captures most of the bioavailable lycopene [32]. In a cohort of healthy young women from the BioCycle Study [33], the authors reported an inverse association between serum lycopene concentration and serum follicle-stimulating hormone (FSH) levels. However, FSH is not considered the best biomarker of ovarian reserve [23], and since these FSH levels were measured at different phases of the menstrual cycle, their comparability with studies of ovarian reserve is limited. In a prospective work that included 1146 premenopausal women from The Melbourne Collaborative Cohort Study, with an average of a 12.5-year follow-up [34], lycopene intake and the consumption of tomato products were positively correlated with the age of natural menopause in unadjusted analyses. Nevertheless, these relationships were no longer statistically significant in the adjusted models, and the late reproductive age of the participants (mean ± standard deviation age at study entry: 46.8 ± 3.1 years) may not be comparable to our study population. In our work, we did not observe any effects of other carotenoids on AFC, although previous studies linked them to indicators of ovarian aging. For example, in a cohort of premenopausal women, β-cryptoxanthin intake from the diet (~400 mcg/day) was associated with a reduction in ovarian senescence by 1.3 years [34].

Among carotenoids, lycopene is considered the most potent single oxygen and free radical scavenger, being more efficient in shielding cells and tissues from damage induced by ROS [35,36]. The antioxidant properties conferred to lycopene include cell growth regulation, gap junction communication, gene expression modulation, immune response, and protection against lipid peroxidation [12]. Although there is a lack of evidence to identify the underlying mechanisms involved in the ovarian reserve, experimental studies in animal models have attributed lycopene with antioxidative effects on ovarian tissue. In rodents, oxidative damage to the ovaries caused by furan [37], cisplatin [38], or methotrexate [21] was reduced with lycopene administration, leading to decreased levels of malondialdehyde (the end-product of lipid peroxidation) and increased levels of antioxidant parameters. The histological examination revealed follicular degeneration in the groups exposed to the toxicant, whereas those treated with lycopene exhibited reduced pathological changes [37] or a normal follicular structure [21,38]. Additionally, treatment with lycopene showed a protective antioxidant capacity against oxidative stress in both naturally aged and induced aged ovaries via the activation of the Nrf2/HO-1 pathway in chickens. The impairment of granulosa cells and growing follicles observed was reduced when treated with lycopene [3]. The depletion of ovarian follicle reserves and the increase in atretic follicles induced by exogenous hormones during multiple cycles of ovarian hyperstimulation (which are known to produce excessive amount of ROS) were mitigated in mice that received lycopene simultaneously. Gene expression analysis further revealed a significantly improved profile of ovarian antioxidant (*Sod3*, *Cat*, *Nrf2*, *Keap1*) and inflammatory (*Tnf*, *Nfkb*, *Casp3*) factors. Additionally, a decreased concentration of H_2_O_2_ was also observed. Lycopene exerted an inhibitory effect on follicular apoptosis, as evidenced by the decreased expression of the apoptosis-related gene *Casp3* in granulosa cells, mainly in those of the secondary order [39]. Given that these were in vitro studies, it is still unclear whether the doses tested are physiologically relevant for humans. To date, none of these mechanisms have been conclusively demonstrated in humans, and animal models or cell-based experimental evidence in this field remains sparse. Thus, the antioxidant impact of lycopene on the ovary does not conclusively equate to safeguarding against a declining ovarian reserve, highlighting the need for further research on this topic.

Although we are not aware of previous works examining the relationship of retinol intake with the makers of ovarian reserve, there is research evaluating this relationship with related outcomes. In four observational studies, of which three were prospective [33,34,40] and one cross-sectional [41], no association was reported between retinol intake [34,40,41] or serum retinol levels [33] and the onset of menopause [34,40,41] and FSH levels [33]. In our study, we observed a strong negative association between retinol intake and AFC, which was only present among participants younger than 35 years of age, suggesting that a window of vulnerability may exist. We had no prior hypothesis for this finding and the biological reasons for this association’s presence in this group of women only are not obvious. A possible explanation for this difference in association for younger and older women could reflect the effect of age itself on ovarian reserve. Given the crucial and well-characterized effect of age on ovarian reserve [42,43], environmental factors, including diet, may have a greater effect on ovarian reserve among younger women than among older women.

Retinol constitutes a dietary precursor of the physiologically active form of all-trans retinoic acid (RA) [44]. While studies indicate that RA supports female reproduction, the role of retinoids in folliculogenesis, including the evaluation of markers of ovarian reserve, has received little attention [44], and findings in the literature are contradictory. Women undergoing treatment with retinoids, such as isotretinoin (13-*cis* retinoic acid), for skin conditions, have experienced significant decreases in ovarian volume [45], AFC [45], and AMH levels [44,45,46,47] and even non-significant differences in ovarian volume [48] and AFC [47,48]. Direct effects of retinoids on ovarian follicles have been observed in rat ovaries, where isotretinoin increased both the percentage of atretic follicles and the number of ovarian follicles with apoptotic granulosa cells [49]. The deleterious effect could be mediated by FoxO1, which is expressed in granulosa cell apoptosis [5,47]. In contrast, RA has been linked with growth-simulating effects on primordial follicles in vitro in an animal model by regulating matrix metalloproteinase expression [18]. It has been argued that varying results may be due to different dose-dependent responses observed throughout studies [44,45]. However, we observed some inconsistencies in our cohort, as total RAE or that from supplements was unrelated to AFC, not even in the top quartiles of consumption, which included women consuming high intakes of retinol above the tolerable upper levels established in this group by the Food and Nutrition Board (3000 mcg/day) [27,50]. Possibly, other factors in retinol food sources, rather than retinol itself, account for the observed association.

Another potential interpretation of our findings regarding retinol intake may be attributed to the consumption of dairy products observed in this study, which aligns with the marginally significant association observed with vitamin A from food sources and AFC, as well as with our previous report [51], where an inverse relationship between the intake of protein from dairy foods and this outcome was observed. This has possibly been attributed to non-nutritive harmful components found in dairy, such as steroid hormones, growth factors, endocrine disruptors, and pesticides [51]. Moreover, a randomized trial in monkeys showed that those fed a dairy protein-based diet (casein–lactalbumin) had significantly fewer primordial, primary, and secondary follicles compared to those on the control diet (soy protein) [52,53], although authors were not able to determine the mechanism for this effect. In addition, a systematic review based on studies in rodent models found that exposure to galactose (a monosaccharide found in dairy) had an ovotoxicity effect on pre-antral and antral follicles, inducing apoptosis in granulosa cells [54]. In contrast to our findings, in a prospective work among eumenorrheic women [9], the authors found that the intake of dairy foods and their constituents (carbohydrates, fat, protein, calcium, lactose, and galactose) reduced the odds of annual and rapid rates of decline in AMH, which was indirectly mediated by lower phosphate, proline, and branched-chain amino acids, as observed in a further metabolomics analysis [55]. We are not aware of studies linking dairy products to ovarian reserve outcomes examining the role of retinol from dairy. Because dairy products are a major source of retinol, but also contain saturated and trans fats, we considered whether these fatty acids might explain the observed associations. While both fatty acids have been associated with inflammatory processes [56], prior evidence specifically linking these fatty acids to ovarian reserve markers is poorly studied, failing to identify associations with ovarian reserve markers among women [9,57]. After further adjustment for these fatty acids, our findings suggest that other unmeasured components in dairy products, as previously mentioned [51], may contribute to these results. It is important to note that our findings for dairy products should be considered exploratory, as we did not establish a pre-defined hypothesis regarding the dairy–retinol–ovarian reserve relationship. Overall, the association pattern observed for retinol and their food sources may suggest that even if these are replicated in other studies, these associations are likely explained by biological mechanisms other than effects on oxidative stress. These results underscore the need for future research, including experimental studies, to disentangle the roles of dietary retinol and its food sources (specifically, related toxic components in dairy products) in ovarian reserve, investigate molecular mechanisms—particularly the effects of oxidative stress on mitochondrial damage and granulosa cell apoptosis—and to characterize the biological pathways underlying our findings.

Several strengths and limitations are worth noting. The FFQ is a self-report method to assess diet and is susceptible to measurement error and misclassification of participants. However, this questionnaire has been extensively validated [24,25], which allowed for a comprehensive assessment of major retinol and carotenoid sources in the U.S. diet. In addition, to further address the potential under- or over-reporting of dietary intakes, which is inherent to self-report-based dietary assessment tools, we also excluded subjects who reported extremely low or high total energy intake in the analysis [58], adjusted nutrient intakes for total energy intake [58], making comparisons between quartiles of intake to provide conservative estimates of the examined associations, restricted the highest retinol intake category to account for high variability limiting intakes below thresholds of two and three standard deviations, and performed additional sensitivity analyses excluding participants above the 95th percentile for the non-linearity analysis. Because some of the antioxidants were moderately to strongly correlated to each other, this could have prevented us from estimating the independent effect on AFC. Conversely, in our statistical strategy, we adjusted for the remaining antioxidants in the final models. We did not include circulating AMH concentrations (another well-accepted ovarian reserve biomarker) as an outcome due to the small sample size. Measurements were routinely performed only starting in 2013, and an assay change during this period made it challenging to combine data over time. Nevertheless, both tests are currently considered reliable in the assessment of ovarian reserve [23]. This work included participants from a fertility clinic, predominantly White (83% vs. 58% in the U.S. Census [59]), with high educational degrees (93% vs. 35% [59]), and had a favorable overall lifestyle profile (e.g., leaner vs. overweight and obesity (32% vs. 74% [60]) and with fewer smokers (2% vs. 11% [60])), which strengthens the internal validity of our results but may not be representative of women from other racial/ethnic minority backgrounds, lower socioeconomic statuses, those with different dietary and lifestyle patterns, or women in the general population. However, our participants closely resemble other fertility patients in the United States, suggesting that the findings may be generalizable to couples undergoing infertility treatment [61]. Diet exposure was documented during the preceding year of the AFC measurement, reflecting participants’ usual intake. Thus, due to our study design, we were unable to examine the long-term dietary habits of the participants and their potential effects on ovarian reserve. Despite this limitation, our dietary assessment likely captured the most relevant time frame for folliculogenesis, a process that requires approximately one year for a primordial follicle to grow and develop before ovulation [62]. Therefore, this assessment may identify dietary effects on the size of the growing follicle pool and, consequently, ovarian reserve. Since the number of growing oocytes is correlated with the primordial pool, effects detected in the growing cohort may also reflect changes in the primordial pool. In our study, we lacked data on OS markers (e.g., ROS, malondialdehyde), which limits our ability to validate the hypothesized mechanisms underlying the observed associations. Finally, as with any observational study, residual or unmeasured confounding, as other lifestyle and diet factors, cannot be completely ruled out despite statistical adjustment for a large number of known and potential confounders. Although our study provides valuable observational data, future randomized controlled trials testing specific interventions based on these findings are needed to establish causality and better understand the clinical implications of these associations.

## 5. Conclusions

In summary, our data from women at a fertility clinic suggest a potential association between lycopene intake and the ovarian reserve, measured as AFC, while no overall associations between the intakes of vitamins A, C, and E and other specific carotenoids examined appeared to be related to this outcome. The inverse association with retinol intake, if confirmed, may reflect biological mechanisms other than effects from oxidative stress. This study contributes to the growing body of research on the role of diet in female fertility and highlights the need for further research to elucidate the potential effects of antioxidants on ovarian reserve.

## Figures and Tables

**Table 1:** Baseline characteristics of 567 women in the EARTH study.

Characteristics			Total
Age (at study entry), years			35 (32–38)
Ever smoked			144 (25.4)
White			470 (82.9)
College degree or higher			527 (92.9)
BMI, kg/m^2^			23.2 (21.2–26.0)
Physical activity, hours/week			5 (2.5–9.9)
Total energy intake, Kcal/day			1647.7 (1335.4–2023.6)
Alcohol, g/day			4.7 (1.4–12.4)
Caffeine, mg/day			103.6 (43.7–171.9)
Folate intake, DFE mg/day			1457.8 (775.8–2095.0)
Vitamin B12 mcg/day			11.3 (8.6–15.6)
Vitamin A, mcg/day			1810.5 (1283.4–2214.8)
Vitamin C, mg/day			174.9 (120.4–236.8)
Vitamin E, mg/day			19.9 (14.3–24.1)
Retinol, mcg/day			1202.5 (794.5–1554.1)
Total carotenoids, mcg/day			14,418.0 (10,747.0–19,274.0)
Alpha-Carotene, mcg/day			549.8 (293.0–933.1)
Beta-Carotene, mcg/day			5816.5 (3802.2–7827.8)
Beta Cryptoxanthin, mcg/day			83.8 (50.9–145.5)
Lycopene, mcg/day			3724.3 (2640.5–5378.3)
Lutein and zeaxanthin, mcg/day			3264.0 (2293.0–4920.3)
Prior pregnancy			250 (44.1)
Prior infertility exam			474 (83.6)
Prior fertility treatment			336 (59.3)
Day 3 FSH, IU/ml			6.9 (5.9–8.4)
Infertility diagnosis			
	Male factor		138 (24.3)
	Female factor		
		DOR	53 (9.3)
		Endometriosis	20 (3.5)
		Ovulatory	54 (9.5)
		Tubal	27 (4.8)
		Uterine	10 (1.8)
	Unexplained		265 (46.7)

BMI: body mass index. DOR: diminished ovarian reserve. FSH: follicle stimulating hormone. Values are presented as medians and interquartile ranges for continuous variables and as absolutes and percentages for categorical variables.

**Table 2:** Association between antioxidant intake and antral follicle count (adjusted mean (95% CI))^ in the EARTH study (*n* = 567).

Antioxidant Intake		Quartile 1	Quartile 2	Quartile 3	Quartile 4	*p*-Trend
Vitamin A **, mcg/day						
*n* (range)		141 (327.44–1279.18)	142 (1283.41–1809.57)	142 (1810.5–2212.89)	142 (2214.77–12,473.66)	
	AFC (95% CI)	13.3 (12.6–14.0)	13.5 (12.8–14.2)	13.1 (12.4–13.8)	13.3 (12.6–14.1)	0.88
Vitamin C, mg/day						
*n* (range)		141 (25.57–120.22)	142 (120.41–174.68)	142 (174.90–236.82)	142 (236.83–1546.70)	
	AFC (95% CI)	12.9 (12.2–13.7)	13.6 (13.0–14.3)	13.6 (12.9–14.4)	13.0 (12.3–13.7)	0.65
Vitamin E, mg/day						
*n* (range)		141 (3.18–14.28)	142 (14.29–19.92)	142 (19.95–24.11)	142 (24.12–354.93)	
	AFC (95% CI)	13.0 (12.3–13.8)	12.8 (12.1–13.5)	13.8 (13.2–14.6)	13.5 (12.7–14.2)	0.21
Preformed retinol, mcg/day						
*n* (range)		141 (100.33–784.86)	142 (794.49–1202.40)	142 (1202.54–1553.03)	142 (1554.05–10,228.47)	
	AFC (95% CI)	13.8 (13.0–14.5)	13.1 (12.5–13.9)	13.6 (13.0–14.4)	12.8 (12.1–13.5) *	0.07
Total carotenoids, mcg/day						
*n* (range)		141 (2428.63–10,713.90)	142 (10,746.57–14,408.07)	142 (14,418.35–19,236.59)	142 (19,274.22–51,287.62)	
	AFC (95% CI)	13.3 (12.6–14.0)	13.3 (12.6–14.0)	13.1 (12.4–13.8)	13.4 (12.7–14.1)	0.79
Alpha-Carotene, mcg/day						
*n* (range)		141 (5.64–292.57)	142 (292.96–549.41)	142 (549.77–931.61)	142 (933.07–4300.58)	
	AFC (95% CI)	13.3 (12.6–14.1)	13.8 (13.0–14.5)	12.8 (12.1–13.5)	13.4 (12.7–14.2)	0.84
Beta-Carotene, mcg/day						
*n* (range)		141 (724.14–3796.19)	142 (3802.24–5801.09)	142 (5816.52–7824.56)	142 (7827.80–30,102.44)	
	AFC (95% CI)	13.0 (12.3–13.8)	13.4 (12.7–14.1)	13.0 (12.3–13.7)	13.9 (13.0–14.8)	0.24
Beta-Cryptoxanthin, mcg/day						
*n* (range)		141 (5.13–50.87)	142 (50.90–83.77)	142 (83.81–145.08)	142 (145.51–693.61)	
	AFC (95% CI)	13.4 (12.8–14.2)	13.0 (12.3–13.7)	13.4 (12.7–14.1)	13.3 (12.7–14.0)	0.91
Lycopene, mcg/day						
*n* (range)		141 (533.19–2622.20)	142 (2640.49–3708.37)	142 (3724.29–5371.89)	142 (5378.27–28,885.46)	
	AFC (95% CI)	13.3 (12.7–14.0)	12.6 (11.9–13.2)	13.6 (12.9–14.3)	13.8 (13.1–14.5)	0.06
Lutein and zeaxanthin, mcg/day						
*n* (range)		141 (565.34–2292.55)	142 (2292.97–3261.47)	142 (3263.99–4902.56)	142 (4920.30–21,623.60)	
	AFC (95% CI)	13.2 (12.5–13.9)	13.4 (12.7–14.0)	13.5 (12.8–14.2)	13.2 (12.5–13.9)	0.82

AFC: antral follicle count. * *p* < 0.05 for comparison of specific quartile vs. quartile 1 (reference). ** Total vitamin A. ^ Adjusted for age, BMI, smoking status, race, physical activity, total energy intake, vitamin B12, folate, caffeine, and intakes of the remaining antioxidants.

## Data Availability

The data underlying this article will be shared on reasonable request to the corresponding and last author.

## Supplementary Materials

The following supporting information can be downloaded at https://www.mdpi.com/article/10.3390/nu17030554/s1: Supplementary Tables S1a,b. Baseline characteristics of women in the EARTH study (*n* = 567) according to quartiles of antioxidant intake; Supplementary Table S2. Spearman correlations between antioxidant intake of 567 women in the EARTH study; Supplementary Table S3. Associations between antioxidant intake (type of intake source) and antral follicle count (adjusted mean (95% CI))^ in participants in the EARTH study (*n* = 567); Supplemental Table S4. Effect modification of the associations between retinol intake and antral follicle count (adjusted mean (95% CI))^ in participants in the EARTH study (*n* = 567). Supplementary Figure S1–S14. Restricted cubic spline plot of the association between antioxidant intake and antral follicle count among 567 women in the EARTH study after multivariable adjustment.
