# Supplementary material for: Antioxidant Intake and Ovarian Reserve in Women Attending a Fertility Center
Source: Nutrients. 2025 Jan 31;17(3):554. doi: 10.3390/nu17030554 (PMC11820690; doi:10.3390/nu17030554)

Supplemental Table 1.a. Baseline characteristics of women in the EARTH study (*n* = 567) according to quartiles of antioxidant intake.

Characteristics	Vitamin A		Vitamin C		Vitamin E		Retinol		Total carotenoids	
	Q1 (lowest)	Q4 (highest)	Q1 (lowest)	Q4 (highest)	Q1 (lowest)	Q4 (highest)	Q1 (lowest)	Q4 (highest)	Q1 (lowest)	Q4 (highest)
n (range)	863.69 (327.44-1279.18)	2502.77 (2214.77-12473.66)	85.09 (25.57-120.22)	318.67 (236.83-1546.7)	9.40 (3.18-14.28)	29.32 (24.12-354.93)	448.31 (100.33-784.86)	1864.30 (1554.05-10228.47)	9044.33 (2428.63-10713.9)	23190.30 (19274.22-51287.62)
n	141	142	141	142	141	142	141	142	141	142
Age (at study entry), years	35 (32-38)	35 (33-38)	35 (31-38)	35 (33-39)	35 (32-38)	35 (32-38)	35 (32-38)	35 (32-37)	35 (32-39)	34.5 (31-38)
Ever smoker	41 (29.1)	35 (24.6)	36 (25.5)	38 (26.8)	43 (30.5)	39 (27.5)	37 (26.2)	34 (23.9)	40 (28.4)	39 (27.5)
White	118 (83.7)	115 (81)	117 (83)	115 (81)	113 (79.6)	117 (82.4)	113 (80.1)	116 (81.7)	116 (82.3)	110 (77.5)
College degree or higher	128 (90.8)	135 (95.1)	130 (92.2)	136 (95.8)	130 (91.5)	133 (93.7)	127 (90.1)	131 (92.3)	125 (88.7)	134 (94.4)
BMI, kg/m2	23.2 (21.2-26)	23.3 (21.2-26.1)	23.6 (21.6-26.1)	22.9 (21.2-25.4)	23.8 (21.5-26.4)	23.2 (21.1-25)	23.3 (21.5-26)	23.5 (21-26.2)	23.3 (21.6-25.7)	23.4 (21.4-25.8)
Physical activity, hours/week	4.5 (1.7-8.7)	6.2 (3-12)*	5 (2.5-9.5)	4.5 (2-9.5)	4.7 (2-8.5)	6.7 (2.9-12.5)	4.6 (1.7-8.7)	5 (2.5-10.5)	5 (1.5-9.5)	5.7 (3.2-11.4)
Total energy intake, Kcal/day	1613.8 (1304.4-2004.5)	1575.8 (1198.2-1879.0)*	1624.1 (1337.8-2041.1)	1652.3 (1374.8-1945.4)	1613.8 (1363.1-2027.1)	1637.0 (1255.9-1915.7)	1617.5 (1324.4-2028.8)	1637.0 (1301.6-2007.8)	1669.2 (1417.1-2004.5)	1745.5 (1411.5-2070.6)
Alcohol, g/day	4.7 (1.4-12.7)	5.0 (1.4-11.6)	4.4 (1.4-10.4)	4.8 (1.2-12.1)	4.6 (1.4-10.8)	5 (1.1-12.4)	4.4 (1.8-12.2)	4.8 (1.1-11.6)	5 (1.4-13.1)	4.9 (1.2-12.7)
Caffeine, mg/day	112.9 (49.4-250.8)	102.3 (24.1-149.9)*	119.1 (49.7-244.9)	100.8 (37.3-154.2)	111.2 (48.7-250.5)	104.5 (38.3-162.5)	119.1 (48.7-239.6)	100.6 (24.1-165.3)	114.8 (39.1-250.5)	105.1 (48.7-165.3)
Folate intake, DFE mg/day	709.3 (383.8-1311.3)	1813.8 (1181.5-2599)**	768.7 (364.2-1331.2)	1998.2 (1250.2-2746.9)**	709.3 (398.1-1311.3)	1671.5 (861.9-2472.8)**	673.4 (367.7-1216.0)	1691.6 (1172.5-2500.8)**	1084.9 (527.5-1475.1)	1535.6 (546.0-2319.4)**
B12 mcg/day	6.1 (4.2-8.7)	13.9 (11.2-21.8)**	6.8 (4.3-9.8)	15.6 (11.6-27.3)**	6.1 (4.2-8.6)	15.3 (11.4-28)**	6.0 (4.2-8.6)	14.4 (10.9-21.8)**	9.6 (6.0-11.6)	12.2 (9.4-16.2)**
Vitamin A, mcg/day	863.7 (645.8-1068.6)	2502.8 (2335.0-2905.1)**	1001.8 (670.7-1313.3)	2022.0 (1699.6-2529.7)**	895.0 (647.8-1176)	2291.0 (1923.5-2819.7)**	863.7 (645.8-1074.8)	2417.0 (2201.8-2864.9)**	1311.7 (781.8-1720.4)	2087.4 (1796.5-2502.7)**
Vitamin C, mg/day	92.7 (65.9-123.9)	221.9 (167.1-299.1)**	85.1 (64.5-105.6)	318.7 (260.4-555.6)**	87.7 (65.9-122.6)	224.7 (174.3-320.6)**	95.0 (68.2-122.6)	192.6 (151.6-290.4)**	121.1 (74.7-162.9)	219.0 (150.0-275.1)**
Vitamin E, mg/day	9.7 (7.3-13.6)	24.8 (22-32.8)**	10.7 (7.9-15.0)	22.8 (18.3-32.2)**	9.4 (7.3-12.2)	29.3 (25.8-37.6)**	10.1 (7.5-13.2)	24.1 (20.6-32.2)**	16.0 (9.4-20.9)	22.7 (16.9-27.6)**
Retinol, mcg/day	464.8 (279.5-647.5)	1821.2 (1529.0-2148.8)**	513.9 (359.2-798.1)	1253.0 (1085.1-1751.4)**	467.7 (305.6-740.9)	1549.8 (1218.9-1996.3)**	448.3 (279.5-588.0)	1864.3 (1681.3-2148.8)**	1015.8 (481.5-1411.6)	1203.2 (911.1-1438.3)*
Total carotenoids, mcg/day	10911.0 (8479.6-14582.0)	18006.0 (14094.0-24495.0)**	10900.0 (8374.7-15323.0)	17248.0 (12723.0-23159.0)**	11231.0 (9116.7-15392.0)	17389.0 (12723.0-22248.0)**	11982.0 (9471.6-17279.0)	14544.0 (11308.0-18066.0)**	9044.3 (7435.5-10024.0)	23190.0 (21118.0-27823.0)**
Alpha-Carotene, mcg/day	389.5 (223.4-658.2)	834.9 (441.1-1367.3)**	397.3 (240.3-694.5)	646.4 (346.1-1124.7)**	482.4 (241.3-751.3)	593.4 (342.5-1137.7)*	491.9 (243.7-770.8)	575.3 (307.0-933.1)	278.1 (204.7-471.3)	925.7 (562.3-1487.6)**
Beta-Carotene, mcg/day	3767.9 (2764.2-5012.3)	8283.5 (5801.1-11267.0)**	3641.1 (2593.1-5494.0)	7586.7 (5580.8-9821.2)**	3818 (2877.1-5985.9)	7579.4 (5403.2-9768.8)**	4144.6 (2926.4-6674.8)	5889.7 (4013.1-8287.7)**	3102.1 (2524.4-3781.5)	9968.6 (7733.4-11761)**
Beta Cryptoxanthin, mcg/day	69.6 (42.4-119.6)	90 (58.2-172.7)*	66.1 (41.5-105.6)	108.4 (62.8-198.6)**	73.3 (42.8-119.6)	95 (56.7-204.0)*	76.1 (48.0-132.9)	86.5 (53.0-138.6)	62.5 (40.5-95.1)	102.3 (60.1-194.0)**
Lycopene, mcg/day	3278.6 (2229.0-4905.6)	3681.1 (2696.2-5127.8)*	3560.8 (2528.3-4963.9)	3681.1 (2609.3-5395.3)	3326.7 (2392.0-4963.9)	3495.6 (2436.9-4900.3)*	3326.7 (2371.8-4905.6)	3545.7 (2622.2-4933.0)*	2815.0 (2030.2-3708.4)	4763.1 (3322.8-8167.4)**
Lutein and Zeaxanthin, mcg/day	2832.9 (1929.1-3783.9)	4231.0 (2699.8-7584.4)**	2641.3 (1766.1-3781.3)	3989.8 (2676.5-7262.9)**	2846.8 (1994.5-4075.9)	4069.1 (2699.8-7373.8)**	3261.5 (2219.9-4650.3)	3113.5 (2246.9-4655.6)	2071.0 (1626.5-2611.8)	7198.8 (4614.7-9832.4)**
Prior pregnancy	70 (49.6)	59 (41.5)	70 (49.6)	56 (39.4)	71 (50.4)	62 (43.7)	72 (51.1)	57 (40.1)	64 (45.4)	65 (45.8)
Prior infertility exam	119 (84.4)	122 (85.9)	115 (81.6)	122 (85.9)	117 (83.0)	119 (83.8)	119 (84.4)	120 (84.5)	112 (79.4)	122 (85.9)
Prior fertility treatment	80 (56.7)	83 (58.5)	81 (57.4)	78 (54.9)	81 (57.4)	80 (56.3)	75 (53.2)	86 (60.6)	87 (61.7)	70 (49.3)*
Day 3 FSH, IU/ml	7.0 (6.0-8.2)	6.8 (6.1-8.0)	6.9 (5.9-8.3)	7.1 (6.0-8.7)	6.8 (5.9-8.2)	6.9 (5.9-8.1)	7.0 (5.9-8.2)	6.6 (6.0-8.0)	6.9 (5.9-8.2)	6.8 (5.9-8.4)
Infertility diagnosis								*		

Male Factor	30 (21.3)	34 (23.9)	26 (18.4)	40 (28.2)	27 (19.1)	34 (23.9)	27 (19.1)	41 (28.9)	37 (26.2)	30 (21.1)
Female factor										
DOR	11 (7.8)	16 (11.3)	12 (8.5)	15 (10.6)	11 (7.8)	14 (9.9)	9 (6.4)	16 (11.3)	14 (9.9)	11 (7.7)
Endometriosis	3 (2.1)	5 (3.5)	4 (2.8)	5 (3.5)	4 (2.8)	4 (2.8)	4 (2.8)	5 (3.5)	3 (2.1)	9 (6.3)
Ovulatory	18 (12.8)	7 (4.9)	17 (12.1)	8 (5.6)	17 (12.1)	16 (11.3)	17 (12.1)	5 (3.5)	7 (5)	13 (9.2)
Tubal	7 (5)	8 (5.6)	4 (2.8)	9 (6.3)	9 (6.4)	6 (4.2)	6 (4.3)	4 (2.8)	10 (7.1)	9 (6.3)
Uterine	3 (2.1)	2 (1.4)	3 (2.1)	2 (1.4)	3 (2.1)	3 (2.1)	2 (1.4)	2 (1.4)	4 (2.8)	2 (1.4)
Unexplained	69 (48.9)	70 (49.3)	75 (53.2)	63 (44.4)	70 (49.6)	65 (45.8)	76 (53.9)	69 (48.6)	66 (46.8)	68 (47.9)

BMI: body mass index. DOR: diminished ovarian reserve. FSH: follicle stimulating hormone. Values are presented as median and interquartile range for continuous variables, absolute and percentage for categorical variables. Kruskal-Wallis and $\chi 2$ test for continuous and categorical variables, respectively: *P-value <0.05, **Significant results after Bonferroni correction for multiple comparison.

Supplemental Table 1.b. Baseline characteristics of women in the EARTH study (*n* = 567) according to quartiles of antioxidant intake.

Characteristics	Alpha-Carotene		Beta-Carotene		Beta-Cryptoxanthin		Lycopene		Lutein and Zeaxanthin	
	Q1 (lowest)	Q4 (highest)	Q1 (lowest)	Q4 (highest)	Q1 (lowest)	Q4 (highest)	Q1 (lowest)	Q4 (highest)	Q1 (lowest)	Q4 (highest)
n (range)	204.71 (5.64-292.57)	1357.67 (933.07-4300.58)	2889.17 (724.14-3796.19)	10076.78 (7827.8-30102.44)	37.3 (5.13-50.87)	224.2 (145.51-693.61)	1985.14 (533.19-2622.2)	7480.3 (5378.27-28885.46)	1733.45 (565.34-2292.55)	7352.65 (4920.3-21623.6)
n	141	142	141	142	141	142	141	142	141	142
Age (at study entry), years	35 (32-39)	35 (32-38)	35 (32-39)	35 (32-38)	34 (31-38)	35 (32-39)	36 (34-39)	35 (32-38)*	35 (32-38)	35 (31-38)
Ever smoker	37 (26.2)	38 (26.8)	37 (26.2)	39 (27.5)	40 (28.4)	27 (19)	39 (27.7)	33 (23.2)	29 (20.6)	32 (22.5)
White	125 (88.7)	106 (74.6)*	124 (87.9)	113 (79.6)	122 (86.5)	100 (70.4)**	108 (76.6)	123 (86.6)	122 (86.5)	110 (77.5)
College degree or higher	129 (91.5)	130 (91.5)	128 (90.8)	134 (94.4)	131 (92.9)	134 (94.4)	126 (89.4)	135 (95.1)	129 (91.5)	136 (95.8)
BMI, kg/m2	23.1 (21.2-25.6)	23.2 (21.2-26.0)	23.1 (21.3-25.6)	23.5 (21.2-25.7)	23.5 (21.2-26.5)	23.2 (21.2-25.1)	22.8 (20.9-25.8)	23.7 (21.3-27.7)	23.7 (21.4-26.1)	23.1 (21.2-24.9)
Physical activity, hours/week	5.0 (2.0-9.0)	5.0 (2.0-10.0)	4.9 (1.5-8.5)	6.5 (3.0-12.0)*	5.0 (2.5-10.7)	5.1 (2.5-9.2)	5.0 (1.5-9.0)	4.8 (2.5-10.2)	4.2 (2.0-9.2)	6.5 (3.5-11.4)*
Total energy intake, Kcal/day	1669.2 (1381.9-2041.1)	1704.9 (1391.9-2027.1)	1617.5 (1337.8-2028.8)	1722.8 (1321.1-2043.1)	1682.3 (1363.1-2028.8)	1750.2 (1310.0-2142.2)	1715.3 (1354.0-2094.1)	1674.7 (1414.5-2142.2)	1657.8 (1398.9-2068.2)	1683.2 (1318.9-2020.4)
Alcohol, g/day	5.0 (2.1-13.5)	3.6 (0.9-11.4)	4.7 (1.2-10.3)	4.7 (1.2-11.6)	5.8 (1.4-13.4)	4.9 (1.4-12.1)	3.8 (1.1-9.9)	4.7 (1.5-12.9)	4.1 (1.1-13.1)	6.2 (1.8-13.5)
Caffeine, mg/day	119.1 (51.4-243.5)	83.4 (26.0-150.6)*	105.2 (36.3-243.4)	102.3 (49.7-157.4)	110.4 (48.1-243.4)	97.0 (25.0-162.8)	106.0 (45.7-215.5)	100.5 (38.3-157.4)	103.2 (43.7-218.6)	104.7 (47.5-165.3)
Folate intake, DFE mg/day	1408.5 (803.4-1936.9)	1535.6 (854.8-2406.5)	1077.3 (609.2-1458.8)	1681.5 (768.7-2579.1)**	1458.8 (824.9-2033.5)	1400.7 (0-2076.1)	1263.1 (543.8-1810.9)	1605.7 (961.5-2303.6)*	1406.3 (805.7-1990.9)	1448.2 (494.7-2063.2)
B12 mcg/day	10.8 (7.8-14.8)	11.8 (9.3-18.6)	9.3 (6.0-11.5)	12.8 (10.4-18.2)**	11.3 (7.9-14.8)	12.2 (9.4-16.4)	11.5 (7.9-16.7)	11.3 (9.0-15.7)	10.8 (7.8-15.2)	12.3 (9.6-18.3)*
Vitamin A, mcg/day	1530.7 (1025.8-1917.8)	2093.4 (1721.0-2493.7)**	1227.3 (671.2-1722.6)	2253.2 (1951.6-2550.4)**	1720.4 (1068.6-2099.4)	1880.1 (1557.8-2275.4)*	1728.9 (1044.0-2201.8)	1813.3 (1468.3-2165.8)	1541.8 (1030.7-1947.9)	2016.5 (1599.8-2432.5)**
Vitamin C, mg/day	144.8 (97.2-211.9)	208.4 (144.2-299.1)**	110.9 (71.1-153.9)	228.2 (167.1-302.6)**	147.6 (90.2-213.7)	209.8 (150.5-265.4)**	154.8 (110.7-243.4)	181.7 (129.1-239.4)	138.9 (87.9-197.4)	215.7 (148.4-275.6)**
Vitamin E, mg/day	19.0 (12.4-22.4)	21.9 (15.8-25.7)*	14.3 (8.9-20.7)	23.5 (20.3-29.2)**	20.3 (12.9-23.3)	20.9 (16.5-25.0)	19.4 (12.4-24.9)	19.2 (15.2-23.7)	17.8 (11.7-21.2)	22.5 (16.9-27.8)**
Retinol, mcg/day	1173.4 (654.7-1518.7)	1199.5 (936.0-1576.9)	982.1 (464.8-1455.4)	1262.9 (1049.6-1619.9)**	1217.3 (748.0-1553)	1210.2 (881.9-1518.7)	1188.5 (611.7-1568.9)	1181.8 (890.6-1470.6)	1182.9 (717.6-1612.7)	1191.4 (869.6-1490.3)
Total carotenoids, mcg/day	10459.0 (8374.7-13023.0)	18999.0 (15698.0-26232.0)**	9311.4 (7439.1-10679.0)	22056.0 (18847.0-26906.0)**	11982.0 (9113.4-16125.0)	17248.0 (13320.0-21228.0)**	11588.0 (8161.5-14746.0)	18074.0 (15467.0-22359.0)**	9842.0 (7593.0-12854.0)	21692.0 (18593.0-26569.0)**
Alpha-Carotene, mcg/day	204.7 (137.1-243.9)	1357.7 (1100.0-1811.4)**	268.9 (189.7-416.6)	1015.8 (615.9-1681.4)**	313.4 (184.5-556.7)	721.8 (389.5-1141.1)**	482.4 (245.2-837.2)	599.0 (361.9-1026.9)	306.5 (215.2-559.4)	799.8 (442.3-1304.6)**

Beta-Carotene, mcg/day	3589.7 (2576.9-5007.4)	8219.7 (6824.4-11122)**	2889.2 (2409.4-3342.8)	10077.0 (8858.5-11761.0)**	4426.9 (3058.9-6830.3)	7002.3 (5404.6-9111.1)**	5374.6 (3575.0-7583.1)	5864.1 (4287.9-7733.4)	3259.3 (2506.4-4665.8)	9188.8 (7318.0-11207.0)**
Beta Cryptoxanthin, mcg/day	54.1 (35.1-100.4)	116.0 (74.4-210.8)**	59.0 (37.3-90.7)	104.9 (64.3-198.6)**	37.3 (28.5-44.5)	224.2 (183.7-305.0)**	79.0 (46.5-141.2)	92.0 (54.1-167.6)	61.3 (39.5-102.0)	113.7 (56.8-194.0)**
Lycopene, mcg/day	3381.4 (2356.5-4386.1)	4041.1 (2728.2-5711.2)*	3560.8 (2484.8-4753.8)	3739.5 (2780.3-4985.3)	3476.1 (2392.0-4704.6)	4079.8 (2699.0-5826.2)	1985.1 (1420.1-2336.0)	7480.3 (5989.4-9221.3)**	3797.4 (2536.4-5395.3)	3861.1 (2728.2-4954.9)
Lutein and Zeaxanthin, mcg/day	2335.1 (1713.6-3296.8)	4332.0 (2964.8-7331.6)**	2025.1 (1562.6-2540.1)	6422.2 (4273.8-9249.4)**	2788.2 (1888.6-3903.2)	4202.3 (2843.6-6543.4)**	3290.0 (2174.5-4696.0)	3354.9 (2279.5-4636.3)	1733.5 (1370.6-2056.4)	7352.7 (5855.1-9832.4)**
Prior pregnancy	62 (44.0)	62 (43.7)	67 (47.5)	66 (46.5)	57 (40.4)	70 (49.3)	68 (48.2)	63 (44.4)	65 (46.1)	55 (38.7)
Prior infertility exam	119 (84.4)	116 (81.7)	110 (78.0)	122 (85.9)	118 (83.7)	122 (85.9)	122 (86.5)	113 (79.6)	114 (80.9)	124 (87.3)*
Prior fertility treatment	85 (60.3)	83 (58.5)	89 (63.1)	71 (50.0)	83 (58.9)	86 (60.6)	86 (61)	92 (64.8)	88 (62.4)	70 (49.3)*
Day 3 FSH, IU/ml	7.2 (6.3-8.3)	7.1 (6.0-8.5)	6.8 (5.9-7.8)	6.8 (5.9-8.4)	6.9 (6.0-8.4)	7.0 (5.9-8.7)	7.2 (6.0-8.7)	7.1 (6.1-8.1)	7.0 (6.0-8.5)	7.0 (6.0-8.5)
Infertility diagnosis										
Male Factor	34 (24.1)	31 (21.8)	37 (26.2)	34 (23.9)	34 (24.1)	35 (24.6)	38 (27.0)	40 (28.2)	43 (30.5)	23 (16.2)
Female factor										
DOR	16 (11.3)	15 (10.6)	13 (9.2)	13 (9.2)	14 (9.9)	16 (11.3)	20 (14.2)	11 (7.7)	17 (12.1)	14 (9.9)
Endometriosis	3 (2.1)	6 (4.2)	5 (3.5)	4 (2.8)	2 (1.4)	4 (2.8)	4 (2.8)	8 (5.6)	2 (1.4)	6 (4.2)
Ovulatory	8 (5.7)	19 (13.4)	11 (7.8)	14 (9.9)	10 (7.1)	14 (9.9)	8 (5.7)	16 (11.3)	13 (9.2)	18 (12.7)
Tubal	8 (5.7)	7 (4.9)	7 (5.0)	6 (4.2)	5 (3.5)	14 (9.9)	11 (7.8)	6 (4.2)	6 (4.3)	8 (5.6)
Uterine	4 (2.8)	0 (0)	5 (3.5)	2 (1.4)	5 (3.5)	1 (0.7)	4 (2.8)	1 (0.7)	3 (2.1)	3 (2.1)
Unexplained	68 (48.2)	64 (45.1)	63 (44.7)	69 (48.6)	71 (50.4)	58 (40.8)	56 (39.7)	60 (42.3)	57 (40.4)	70 (49.3)

BMI: body mass index. DOR: diminished ovarian reserve. FSH: follicle stimulating hormone. Values are presented as median and interquartile range for continuous variables, absolute and percentage for categorical variables. Kruskal-Wallis and $\chi 2$ test for continuous and categorical variables, respectively: *P-value <0.05, **Significant results after Bonferroni correction for multiple comparison.

Supplemental Table 2. Spearman correlations between antioxidant intake of 567 women in the EARTH study.

	Vitamin A	Vitamin C	Vitamin E	Retinol	Alpha-Carotene	Beta-Carotene	Beta Cryptoxanthin	Lycopene	Lutein and Zeaxanthin
Vitamin A	1	0.56	0.71	0.89	0.32	0.55	0.13	0.06	0.30
Vitamin C		1	0.54	0.46	0.22	0.48	0.25	0.07	0.29
Vitamin E			1	0.66	0.14	0.41	0.09	0.02	0.26
Retinol				1	0.05	0.19	0.00	0.03	0.01
Alpha-Carotene					1	0.62	0.37	0.10	0.38
Beta-Carotene						1	0.30	0.06	0.74
Beta-Cryptoxanthin							1	0.06	0.27
Lycopene								1	0.02
Lutein and Zeaxanthin									1

Vitamin A: Retinol activity equivalents.

Supplemental Table 3. Associations between antioxidant intake (type of intake source) and antral follicle count (adjusted mean (95% CI))^ in participants in the EARTH Study (*n* = 567).

Antioxidant intake	Q1	Q2	Q3	Q4	P-trend
**Vitamin A, mcg/day**					
From food, n (range)	141 (286.80-640.94)	142 (641.00-814.45)	142 (817.74-1020.25)	142 (1020.99-2633.30)	
AFC (95% CI)	13.6 (12.9-14.4)	13.2 (12.6-13.9)	14.2 (13.4-14.9)	12.3 (11.7-13.1)*	0.05
From supplements, n (range)	142 (0-428.57)	161 (428.57-900.00)	136 (900.00-1200.00)	128 (1200.00-11400.00)	
AFC (95% CI)	13.3 (12.6-14)	13.7 (13.0-14.3)	12.7 (12.0-13.4)	13.5 (12.7-14.2)	0.81
**Vitamin C, mg/day**					
From food, n (range)	141 (18.85-65.29)	142 (65.34-86.19)	142 (86.30-116.24)	142 (116.27-341.55)	
AFC (95% CI)	13.1 (12.4-13.8)	12.7 (12.0-13.4)	14.1 (13.4-14.8)*	13.2 (12.5-13.9)	0.44
From supplements, n (range)	141 (0-34.28)	142 (34.28-68.40)	156 (68.40-120.00)	128 (120.00-1120.04)	
AFC (95% CI)	13.0 (12.3-13.7)	13.5 (12.8-14.2)	13.5 (12.8-14.2)	13.2 (12.5-14.0)	0.81
**Vitamin E, mg/day**					
From food, n (range)	141 (2.83-6.47)	142 (6.47-8.19)	142 (8.20-10.72)	142 (10.73-40.97)	
AFC (95% CI)	13.5 (12.9-14.2)	13.3 (12.7-14.0)	13.6 (12.9-14.3)	12.6 (11.9-13.4)	0.06
From supplements, n (range)	141 (0-4.97)	131 (5.24-12.67)	150 (13.50-13.50)	145 (13.50-282.44)	
AFC (95% CI)	13.0 (12.3-13.8)	13.1 (12.4-13.9)	13.0 (12.4-13.7)	14.0 (13.3-14.8)	0.27
**Beta-Carotene, mcg/day**					
From food, n (range)	141 (736.39-3041.05)	142 (3048.49-4592.69)	142 (4597.94-6642.98)	142 (6658.25-22403.31)	
AFC (95% CI)	13.6 (12.8-14.4)	12.3 (11.7-13.0)*	13.2 (12.5-13.9)	14.2 (13.2-15.2)	0.13
From supplements, n (range)	140 (0-137.14)	143 (150.00-428.58)	143 (428.58-1800.00)	141 (1800.00-19799.99)	
AFC (95% CI)	12.8 (12.1-13.6)	13.7 (13.0-14.4)	13.4 (12.7-14.2)	13.3 (12.6-14.1)	0.94

AFC: antral follicle count. *P <0.05 for comparison of specific quartile vs quartile 1 (reference). ^Adjusted for age, BMI, smoking status, race, physical activity, total energy intake, vitamin B12, folate, caffeine, and intakes of the remaining antioxidants.

Supplemental Table 4. Effect modification of the associations between retinol intake and antral follicle count (adjusted mean (95% CI))^ in participants in the EARTH study (*n* = 567).

Antioxidant intake	Q1	Q2	Q3	Q4	P-interaction
**Age**					
<35 years, n (range)	66 (154.2-784.9)	56 (796.1-1202.4)	66 (1202.5-1553.0)	63 (1554.6-10228.5)	
AFC (95% CI)	17.3 (16.2-18.5)	15.6 (14.6-16.8)*	16.0 (15.0-17.1)	14.2 (13.3-15.3)*	
≥35 years, n (range)	75 (100.3-780.9)	86 (794.5-1202.1)	76 (1207.5-1546.6)	79 (1554.1-8886.8)	
AFC (95% CI)	11.5 (10.7-12.4)	11.4 (10.6-12.2)	12.3 (11.5-13.2)	11.9 (11.1-12.8)	<0.001
**BMI**					
<25 Kg/m^2, n (range)	92 (129.9-780.9)	96 (794.5-1200.0)	105 (1202.5-1553.0)	94 (1554.1-10228.5)	
AFC (95% CI)	14.0 (13.1-14.9)	13.6 (12.8-14.5)	13.2 (12.5-14.0)	12.9 (12.1-13.8)	
≥25 Kg/m2, n (range)	49 (100.3-784.9)	46 (810.1-1202.4)	37 (1204.0-1546.0)	48 (1554.6-3890.8)	
AFC (95% CI)	13.4 (12.4-14.5)	12.3 (11.3-13.4)	15.2 (13.9-16.5)*	12.6 (11.6-13.7)	0.34
**Smoking Status**					
Never smoker, n (range)	104 (100.3-784.9)	104 (794.5-1202.1)	107 (1202.5-1553.0)	108 (1554.1-10228.5)	
AFC (95% CI)	14.3 (13.5-15.1)	13.7 (12.9-14.5)	14.5 (13.7-15.3)	13.1 (12.3-13.8)*	
Ever smoker, n (range)	37 (144.9-780.9)	38 (796.1-1202.4)	35 (1212.4-1535.3)	34 (1554.6-8511.4)	
AFC (95% CI)	13.2 (12.0-14.6)	12.6 (11.5-13.9)	12.6 (11.5-13.9)	13.1 (11.9-14.5)	0.47

AFC: antral follicle count. *P <0.05 for comparison of specific quartile vs quartile 1 (reference).

^Adjusted for age, BMI, smoking status, race, total energy intake, vitamin B12, folate, caffeine, and intakes of the remaining antioxidants.

Supplementary Figure 1. Restricted cubic spline plot of the association between vitamin A intake and antral follicle count among 567 women in the EARTH Study after multivariable adjustment. P-value for non-linearity = 0.25.

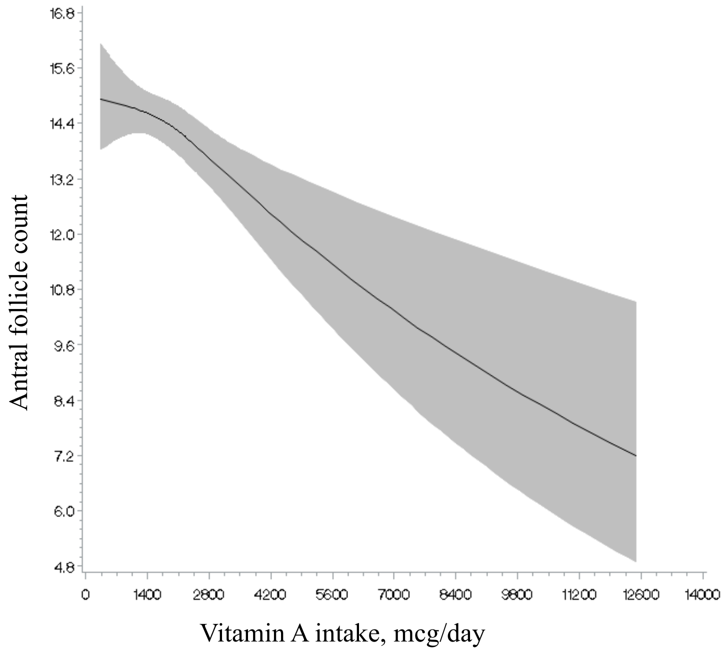

Supplementary Figure 2. Restricted cubic spline plot of the association between vitamin C intake and antral follicle count among 567 women in the EARTH Study after multivariable adjustment. P-value for non-linearity = 0.76.

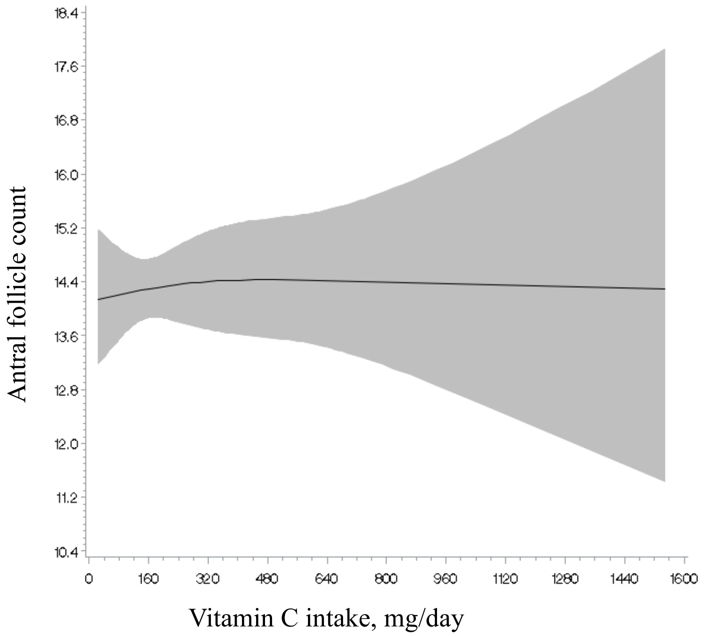

Supplementary Figure 3. Restricted cubic spline plot of the association between vitamin E intake and antral follicle count among 567 women in the EARTH Study after multivariable adjustment. P-value for non-linearity = 0.39.

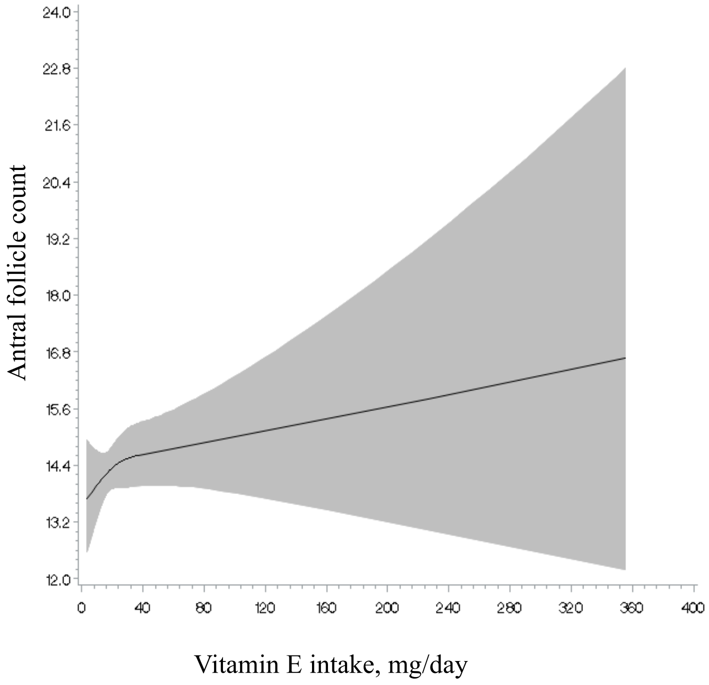

Supplementary Figure 4. Restricted cubic spline plot of the association between total carotenoids intake and antral follicle count among 567 women in the EARTH Study after multivariable adjustment. P-value for non-linearity = 0.40.

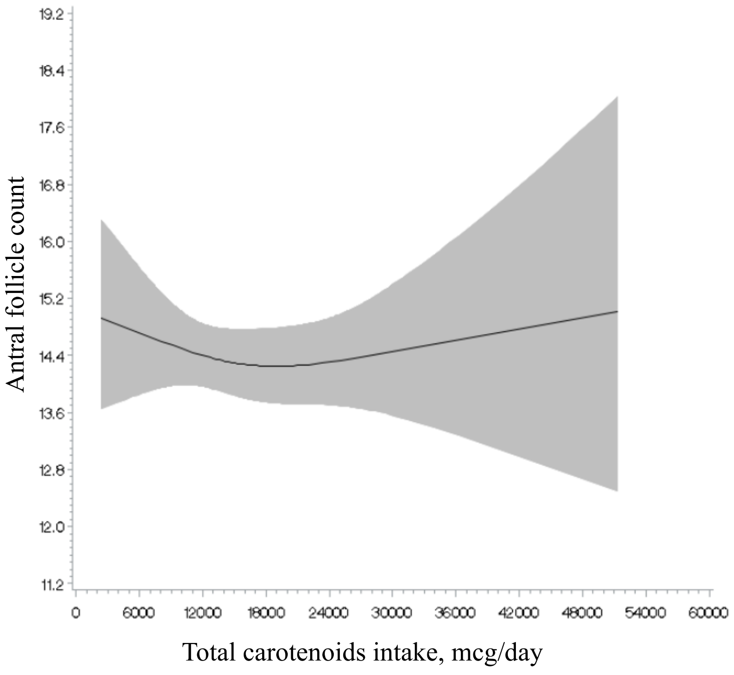

Supplementary Figure 5. Restricted cubic spline plot of the association between Alpha-Carotene intake and antral follicle count among 567 women in the EARTH Study after multivariable adjustment. P-value for non-linearity = 0.75.

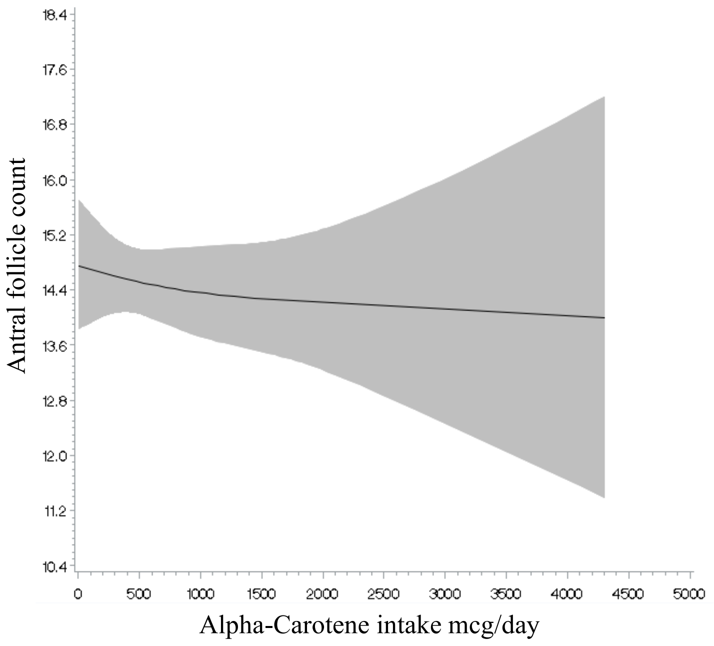

Supplementary Figure 6. Restricted cubic spline plot of the association between Beta-Carotene intake and antral follicle count among 567 women in the EARTH Study after multivariable adjustment. P-value for non-linearity = 0.55.

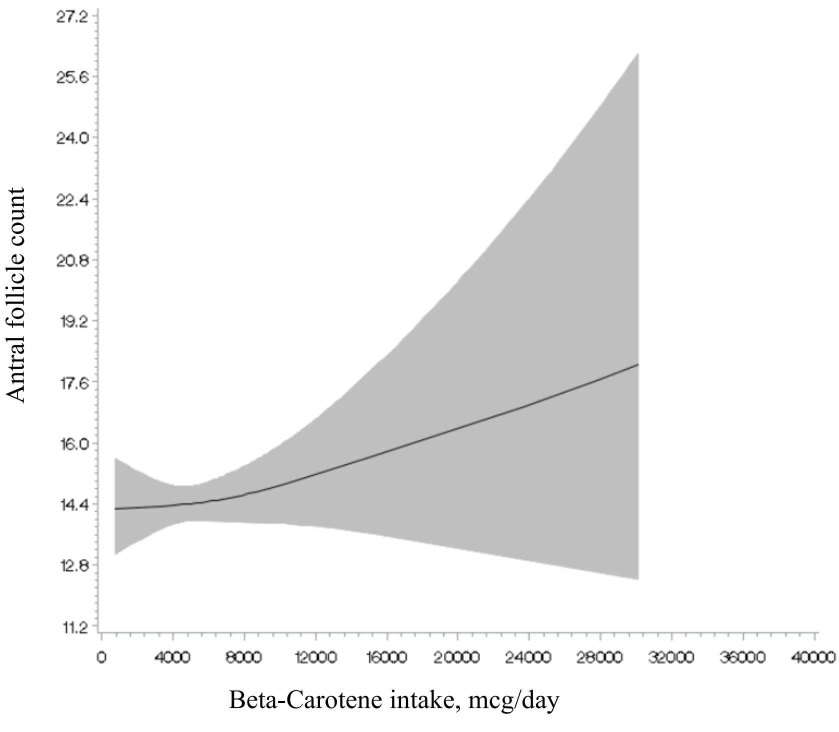

Supplementary Figure 7. Restricted cubic spline plot of the association between Beta Cryptoxanthin intake and antral follicle count among 567 women in the EARTH Study after multivariable adjustment. P-value for non-linearity = 0.41.

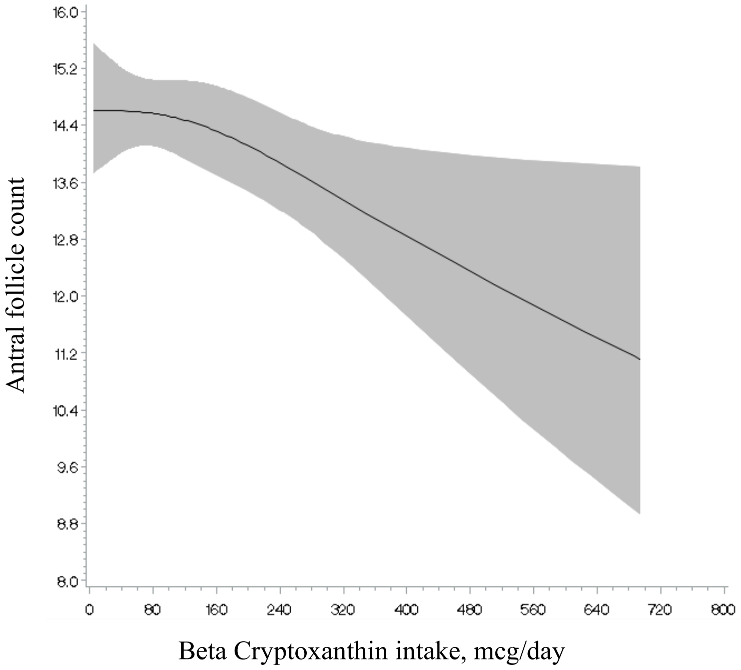

Supplementary Figure 8. Restricted cubic spline plot of the association between lutein and zeaxanthin intake and antral follicle count among 567 women in the EARTH Study after multivariable adjustment. P-value for non-linearity = 0.51.

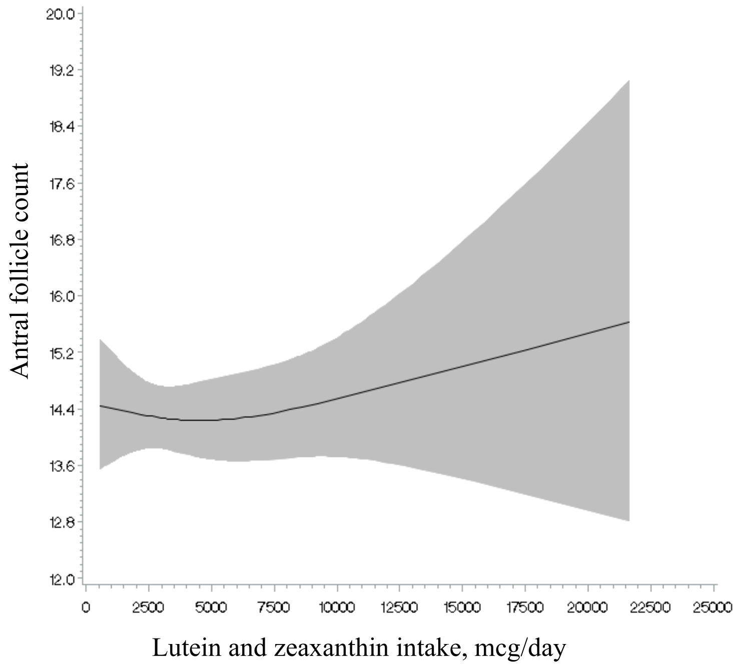

Supplementary Figure 9. Restricted cubic spline plot of the association between vitamin A intake (food source) and antral follicle count among 567 women in the EARTH Study after multivariable adjustment. P-value for non-linearity = 0.97.

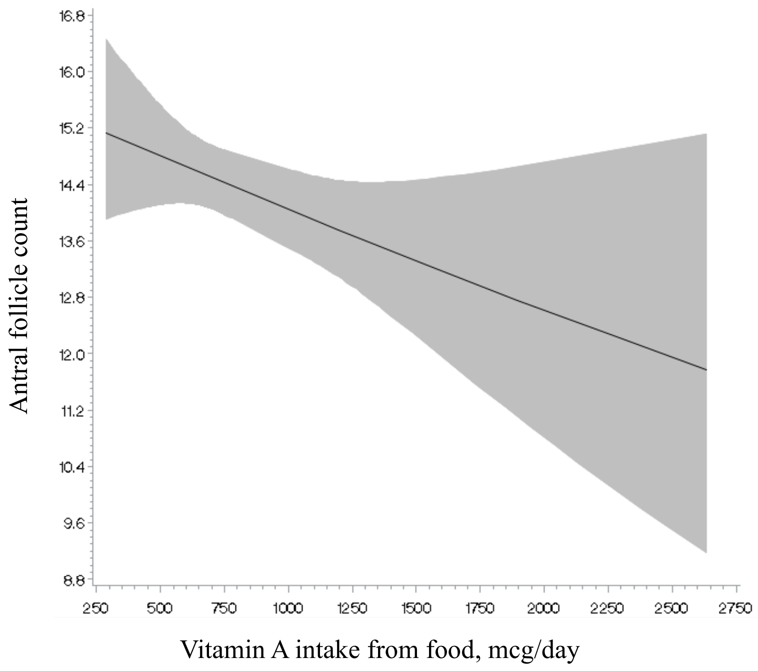

Supplementary Figure 10. Restricted cubic spline plot of the association between vitamin E intake (food source) and antral follicle count among 567 women in the EARTH Study after multivariable adjustment. P-value for non-linearity = 0.04. After restriction at the 95th percentile of the vitamin E intake from food distribution, the P-value for non-linearity was 0.23.

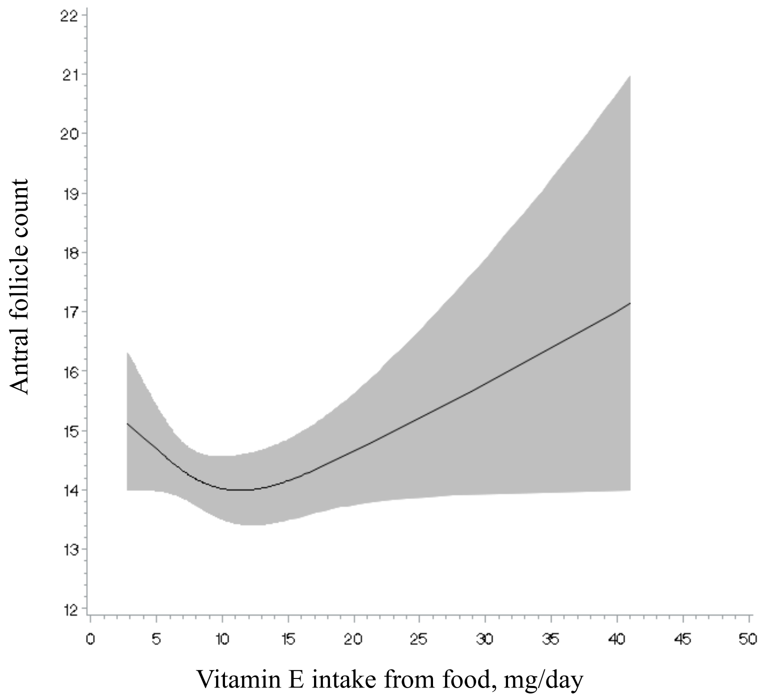

Supplementary Figure 11. Restricted cubic spline plot of the association between Beta-Carotene intake (food source) and antral follicle count among 567 women in the EARTH Study after multivariable adjustment. P-value for non-linearity = 0.25.

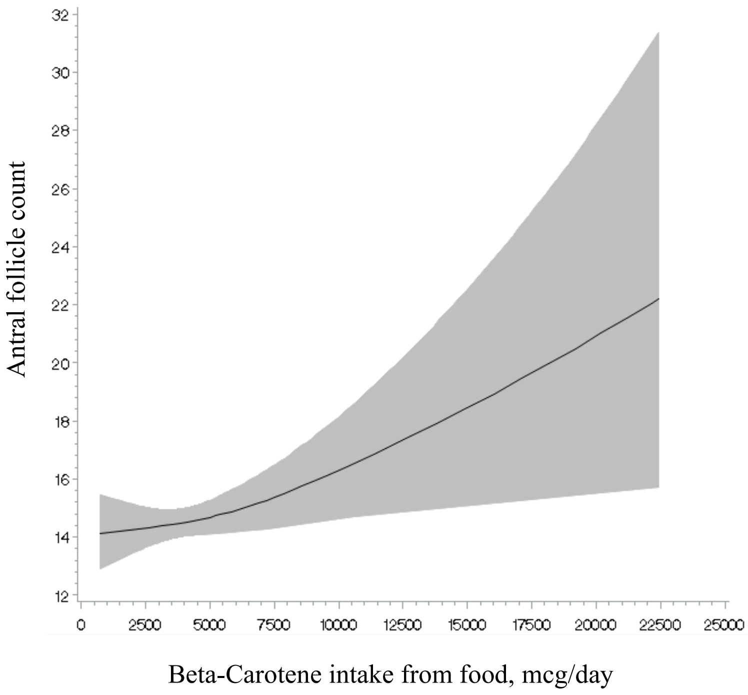

Supplementary Figure 12. Restricted cubic spline plot of the association between vitamin A intake (supplement source) and antral follicle count among 567 women in the EARTH Study after multivariable adjustment. P-value for non-linearity = 0.15.

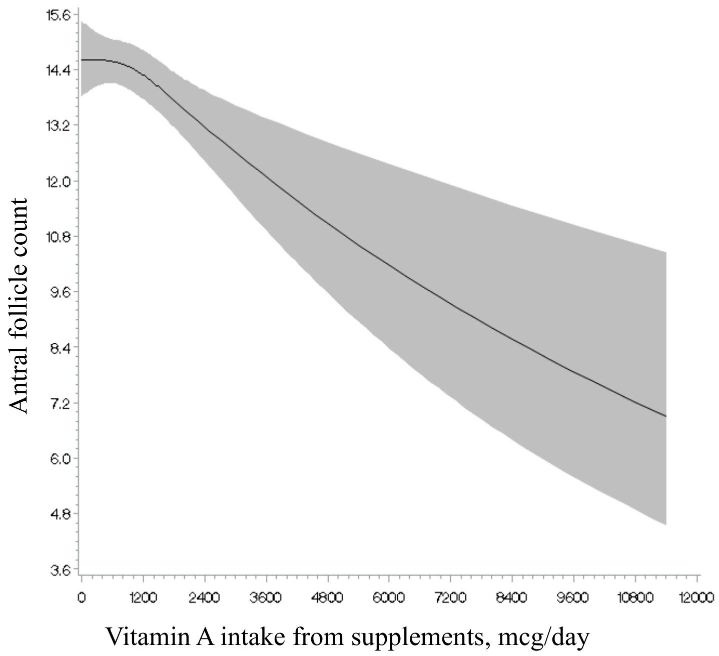

Supplementary Figure 13. Restricted cubic spline plot of the association between vitamin E intake (supplement source) and antral follicle count among 567 women in the EARTH Study after multivariable adjustment. P-value for non-linearity = 0.24.

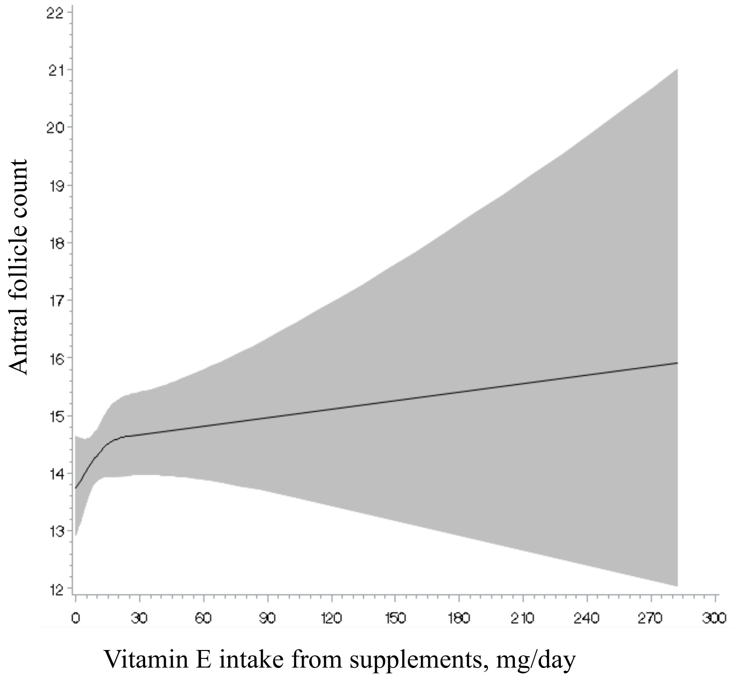

Supplementary Figure 14. Restricted cubic spline plot of the association between Beta-Carotene intake (supplement source) and antral follicle count among 567 women in the EARTH Study after multivariable adjustment. P-value for non-linearity = 0.18.

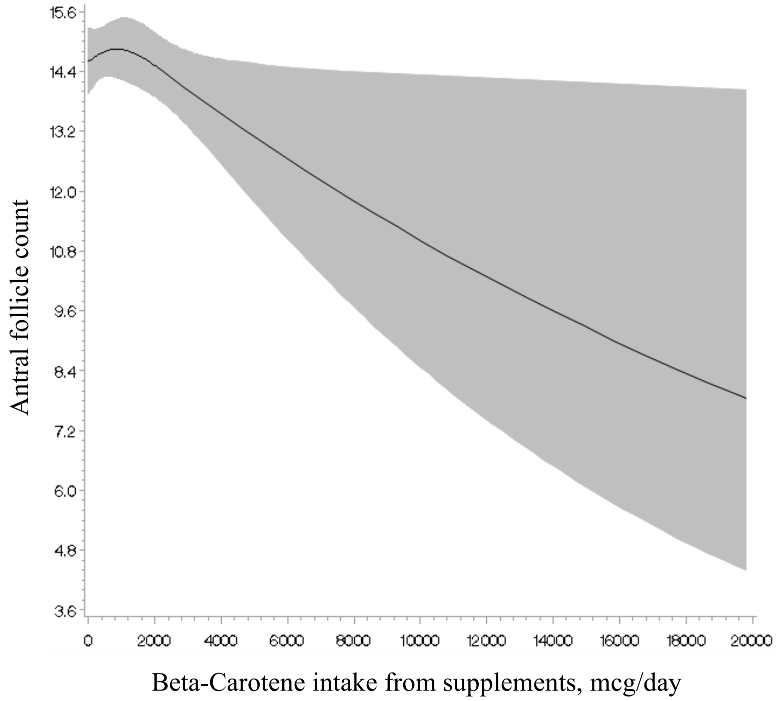

Supplement: Supplementary file 1 [file nutrients-17-00554-s001.zip › nutrients-3417543-supplementary.pdf]
